# Rural Residents’ Perspectives on an mHealth or Personalized Health Coaching Intervention: Qualitative Study With Focus Groups and Key Informant Interviews

**DOI:** 10.2196/18853

**Published:** 2021-02-26

**Authors:** Nancy Schoenberg, Madeline Dunfee, Hannah Yeager, Matthew Rutledge, Angela Pfammatter, Bonnie Spring

**Affiliations:** 1 Department of Behavior Science University of Kentucky Lexington, KY United States; 2 University of Rochester Rochester, NY United States; 3 Department of Statistics University of Kentucky Lexington, KY United States; 4 Department of Preventive Medicine Northwestern University Chicago, IL United States

**Keywords:** rural populations, technology, exercise, diet, community-based participatory research, mobile phone

## Abstract

**Background:**

Compared with national averages, rural Appalachians experience extremely elevated rates of premature morbidity and mortality. New opportunities, including approaches incorporating personal technology, may help improve lifestyles and overcome health inequities.

**Objective:**

This study aims to gather perspectives on whether a healthy lifestyle intervention, specifically an app originally designed for urban users, may be feasible and acceptable to rural residents. In addition to a smartphone app, this program—Make Better Choices 2—consists of personalized health coaching, accelerometer use, and financial incentives.

**Methods:**

We convened 4 focus groups and 16 key informant interviews with diverse community stakeholders to assess perspectives on this novel, evidence-based diet and physical activity intervention. Participants were shown a slide presentation and asked open-ended follow-up questions. The focus group and key informant interview sessions were audiotaped, transcribed, and subjected to thematic analysis.

**Results:**

We identified 3 main themes regarding Appalachian residents’ perspectives on this mobile health (mHealth) intervention: personal technology is feasible and desirable; challenges persist in implementing mHealth lifestyle interventions in Appalachian communities; and successful mHealth interventions should include personal connections, local coaches, and educational opportunities. Although viewed as feasible and acceptable overall, lack of healthy lifestyle awareness, habitual behavior, and financial constraints may challenge the success of mHealth lifestyle interventions in Appalachia. Finally, participants described several minor elements that require modification, including expanding the upper age inclusion, providing extra coaching on technology use, emphasizing personal and supportive connections, employing local coaches, and ensuring adequate educational content for the program.

**Conclusions:**

Blending new technologies, health coaching, and other features is not only acceptable but may be essential to reach vulnerable rural residents.

## Introduction

### Overview

This paper describes the perceptions of Make Better Choices 2 (MBC2), a multi-component diet and physical activity intervention. Although MBC2 has been shown to improve outcomes in urban population, the program has never been implemented among rural residents [1]. In part, this lack of implementation stems from the assumed limited acceptability of, access to, and use of technology, which forms a core component of MBC2. With the growing use of smartphones and other technologies, rural residents may be well-positioned to benefit from this intervention. The purpose of this study is to better understand the perceived feasibility and acceptability of the mobile health (mHealth) intervention and adaptations that should be made to improve fit among the rural Appalachian community before implementing the intervention. Qualitative approaches are well suited to identify perceptions of feasibility, acceptability, and adaptation needed for mHealth programs [2].

We convened 38 participants in 4 focus groups, complemented by 16 key informant interviews, with peer debriefing through a 10-person community advisory board [3]. Using the Consolidated Framework for Implementation Research (CFIR) [4], we describe participants’ overall assessments, including perceived feasibility and acceptability, potential challenges, and required modifications. It should be noted that the program of focus, MBC2, is a multiple-component intervention using mHealth and other elements (health coaching, accelerometers, and incentives) rather than an exclusively mHealth intervention. Although these other components of MBC2 have been successfully deployed and described in this population, mHealth remains under examination among rural residents. Thus, we focus on the personal technology component of the intervention rather than on other components.

### Setting and Background

Rural residents, including those from the central Appalachian region (Kentucky, North Carolina, Tennessee, and West Virginia), experience some of the nation’s greatest resources and health burdens [5]. Challenging conditions include low personal and community-level resources (eg, minimal public transportation and health care professional shortages) [6]. For example, the median household income (US $33,492) in Appalachian Kentucky is overall US $20,000 lower than the United States (US $53,889) median household income. Nearly three-quarters of Appalachian Kentucky counties are classified as *economically distressed*, with economic indicators in the lowest 10% of all US counties [7]. In addition, in many communities throughout Appalachia, few supermarkets, sparse public health and physical activity programming, and inadequate transportation reduce access to high quality, affordable food and recreational opportunities [8].

Associated with these community and personal resource challenges, rural Appalachian Kentucky residents have among the worst health profiles in the United States [8], including elevated rates of cancer, cardiovascular disease, diabetes, and other leading causes of mortality [5,8,9]. These health conditions are mediated by lifestyle behaviors, including suboptimal diet and physical inactivity [6,10]. As a result of these high rates of risk factors and morbidity, life expectancy in the region has been decreasing over the past two decades. Of the 10 counties in the United States experiencing the greatest decline in life expectancy, 8 are located in Appalachia [5]. This alarming trend of suboptimal and worsening health status requires new and innovative approaches, including leveraging technology that may overcome sparse community and personal resources.

### Increasing Use of Technology

The World Health Organization has described mHealth systems as a “medical and public health practice supported by mobile devices, such as mobile phones, patient monitoring devices, personal digital assistants, and other wireless devices” [11]. As applied to personal technology, mHealth may refer to “health and medical prevention and treatment supported by exponential technologies, including but not limited to wireless gateways and connectivity, biosensors and wearable personal technology, precision medicine, and of course, patient engagement and empowerment” [12]. Although personal technology approaches to improving lifestyle have been tested in traditionally underserved urban populations, the feasibility and impact of mHealth interventions among vulnerable rural populations have been largely unexplored [13]. In some rural locations, inconsistent mobile phone reception [14], lack of smartphone ownership [15], and concerns about inadequate internet connectivity and costs have been thought to limit the implementation of mHealth interventions.

New evidence suggests that rural residents, including those from rural Appalachia, increasingly use and are favorably oriented toward personal technology [16]. A recent Robert Wood Johnson Foundation, National Public Radio, and Harvard TH Chan School of Public Health poll reported that 85% of rural residents use the internet, nearly 70% of whom use the internet to obtain health information [17]. Although access problems persist—21% and 10% of rural residents indicate that internet connectivity constitutes a problem or a major problem, respectively [18]—the vast majority of rural dwellers view technology as vital to compensate for sparse or absent community resources. Indeed, most people in the region have smartphones (68%), have home internet access, and use the internet (78%) [18].

mHealth offers distinct promise for special populations [2] such as rural residents, including the potential to engage in programs while overcoming the challenges of long-distance travel, sparse community programming, community peer pressure, and few health care professionals. Providing health and wellness services remotely through phone or internet capabilities offers a potentially lower cost option that circumvents limited transportation options [19,20]. At the same time, research suggests that mHealth intervention effectiveness largely depends on tailoring the program to the preferences of the target group [21]. Thus, additional research is needed to establish the optimal fit among resident preferences and community capacity to produce effective interventions [21]. As our intention was to assess the potential feasibility and acceptability—the first step in intervention administration—our findings reflect theoretical and not experiential perspectives with the MBC2 program. Thus, this study aims to obtain insights into the perceived feasibility and acceptability of mHealth interventions and adaptations that should be made to improve fit among the rural Appalachian community.

## Methods

### Research Approach

Qualitative approaches have long been used to conduct pilot research on perceived intervention feasibility and acceptability; indeed, qualitative research has become the standard approach to initiating many intervention protocols [22]. Thus, to assess the perspectives of rural Appalachian adults on the acceptability and feasibility of employing mHealth interventions, this qualitative study employed focus groups and key informant interviews. We used both focus groups and key informant interviews because each method has the capacity to reveal distinct insights. During the 4 focus groups, diverse community representatives were shown slides and asked semistructured questions about the multiple-component, targeted lifestyle mHealth program, MBC2. During the 16 key informant interviews, the same slide deck was shown to individuals with specific expertise who were queried about how MBC2 program components might fit with their community and clients.

### Setting and Sample

This study was conducted in 6 rural counties in Appalachian Kentucky. The counties were selected because they share many features with the broader group of 54 Appalachian Kentucky counties and Central Appalachia, including access to health and social services, internet and technology use, economic status, and rurality [9]. We used rural-urban commuting area (RUCA) codes to determine the extent of rurality within these counties. Participants resided in counties with RUCA codes ranging from 7 (nonmetro, urban population of 2500-19,999, not adjacent to a metro area) to 9 (nonmetro, completely rural or less than 2500 population, not adjacent to a metro area) [23]. Our campus-community 16-year partnership working in these counties facilitated all aspects of the research conducted.

#### Focus Groups

To obtain a broad array of perspectives and local relevance, we used convenience sampling [24], overcoming potential limits to inclusivity by employing maximum variation sampling [25]. The focus group participants’ personal characteristics (education, income, and lifestyle behaviors) were similar to the central Appalachian population overall and were more likely to be representative of the potential intervention participants, whereas the key informants provided insights based on their specific background and expertise (ie, as a parent, an older adult, a tech sector worker, or a minimal user of technology). Although participants from both groups had a higher socioeconomic status than the average county resident, they were highly representative of potential participants in the intervention [7], which is of greater relevance to this study.

Our local project coordinators contacted community stakeholders in these rural Appalachian counties and requested their participation in focus groups and interviews. These contacts were made informally through their workplaces, churches, and social service agencies. Eligibility criteria included being an adult (age 18 years and above), Appalachian resident, and willingness to participate. Focus groups and key informant interviews were conducted in a wide array of settings (eg, local libraries, the US Department of Agriculture’s Cooperative Extension Service, community center, and government building), at various times of the day (to encourage participation by working people, parents, and older adults), and among diverse stakeholders (varied ages and ethnicity or race, both sexes, and differing employment status) to ensure inclusivity across demographic characteristics. The focus groups included 6-10 participants. We conducted 4 focus groups dictated by theoretical saturation [26], known as the point at which adding new data does not substantially contribute to thematic development or insights.

#### Key Informant Interviews

Consistent with the intention of key informant interviews, we aimed to have special expertise or background represented through our key informant interviews and used purposive sampling [27]. Purposive sampling involves identifying individuals who maintain relevant knowledge or experience on a focal issue and who are willing and capable of engaging in the research [27]. Our key informants included representatives of targeted organizations, including churches, worksites, and community centers. Local project coordinators sought individuals in their personal networks who were more likely to represent the potential intervention participants. In contrast, the key informants provided insights based on their specific backgrounds and expertise (ie, as a parent, an older adult, a tech sector worker, or a minimal user of technology). Eligibility criteria included being an adult (age 18 years and above), Appalachian resident, and willingness to participate. As the MBC2 program enrolls eligible adults of any age, we aimed to explore perspectives from all ends of the age spectrum. Both focus group and key informant interview sample sizes were dictated by theoretical saturation [26], known as the point at which adding new data does not substantially contribute to thematic development or insights. After 16 key informant interviews, we reached theoretical saturation and completed data collection.

### Human Subjects Protection

All study procedures were approved by the Institutional Review Board of the University of Kentucky, protocol #4791. Before enrollment in the study, rights and responsibilities associated with this project were explained to each participant. Interviewers, all of whom had successfully completed the Collaborative Institutional Training Initiative in human subjects’ protection training, responded to questions or concerns. The interviewer and participant then signed human subject protection documents, and a copy was left with each party.

### Data Collection

Upon completion of informed consent documentation, the focus group moderator (a qualitative researcher with 2 decades of focus group experience) described the session, asked an ice-breaker question, and initiated the slide show displaying all program components. Textbox 1 (Note: focus group guides are similar but brief and more general) shows the questions asked during our focus groups.

These questions were developed according to principles of participatory action research, where stakeholders and researchers work together to develop research protocols [28]. Stakeholders included a community advisory board that was convened for this specific project and included 11 local residents from 4 counties. To establish these questions, the researchers grounded the questions in the domain and constructs of the CFIR. CFIR domains and constructs included the individual (eg, self-efficacy), intervention characteristics (eg, complexity), the outer and inner setting (eg, relative priority, available resources, and incentives), and the process of administering the intervention (eg, champions and engagement). During a community advisory board meeting, we presented questions one at a time to obtain feedback on the wording, clarity, and flow. Once these CFIR-based constructs were transformed into questions by stakeholders (the community advisory board and researchers), the interview guide and the slide show were vetted, pilot tested with 6 new local residents, and revised in vivo. To further ensure fit and cultural consonance, we engaged the community advisory board in a second confirmatory round of reviews and we revised accordingly. Finally, in accordance with the principles of participatory action research, we further revised the interview guide after our first focus group.

The moderator employed prompts and ensured that all of those attending provided input; 2 observers took extensive notes, and the session was audio-recorded after participants provided verbal consent. The focus group sessions lasted 70-90 minutes. For the key informant interviews, upon arranging a mutually agreeable meeting time and location (a participant’s home or a community site like a library), interviewers administered informed consent protocols. The interview proceeded with an interview guide based on CFIR but tailored to the particular expertise represented. For example, a technology expert received questions about the standard data use patterns in the region. Each key informant interview lasted 45-60 minutes. For both the focus groups and interviews, participants filled out a pencil and paper questionnaire with standard and validated demographic and relevant health behaviors [29,30] (Table 1). Participants were compensated US $50, a standard honorarium in this region when transportation reimbursement is not provided.

**Sample interview guide for key informant interview based on the Consolidated Framework for Implementation Research. Note: focus group guides are similar but brief and more general.**

Intervention characteristics:

- If we train people on the app, can they use it on their own?
- For the personal coaching, who would be best in this role?
  Trained local people?
- The program takes 6 weeks. You use the app a few times a day, and you use an accelerometer to measure your physical activity. What do you think?
  Are people willing to do this?
  Is this too much?
  Not enough to change people’s lifestyle?
- What do you think the main barriers to starting the program would be?
- Why do you think people would drop out?
- Do you think the program would fit into peoples’ lives?

Outer setting:

- If people want to eat more fresh fruits and vegetables, where do they go to buy these things?
- Are there programs to help people with lower income afford fresh foods?
- What kinds of physical activities are the most popular? Walking? Biking? Fitness classes?
- Where do people exercise? Inside the home? Gym? Outside (school track or parks)? Churches? Schools?

Internet:

- Are people willing to use their data for this app?
- How often do you think people change their internet provider or phone? Why do people make changes? Is cost a factor?
- Do you think people regularly experience interruptions in service when trying to upload or download data?
- Are there places where people can access free Wi-Fi, like in a library?

Exercise:

- Are fitness club memberships expensive?
  Are fees determined based on a person’s income?
- Are there programs to help people with lower income afford memberships?
- Do places for recreation have childcare?
- Are places for recreation open all the time or are there certain hours?

Inner setting:

-
  Do you think that we would find people who could be trained as personal coaches? Where? Who would be good?
  What would be the best approach for training these personal coaches?
  Are there any logistical issues that we should remember?
- This is a home-based program, so people would not have to travel except for the assessments (baseline and 3, 6, and 9 months).
  How should we best communicate with our participants? Facebook, email, telephone, and visit?

Individuals’ characteristics:

- If we want to get a fuller range of people involved, how can we do that?
  For example, how do we get men to participate in the program?
- How do we get those people who are not particularly motivated to join?
  How about those people who think they cannot change their lifestyle?

Implementation process:

- How can we make sure that our coaches deliver personal coaching in the same way to all participants?
- How can we make sure that the participants are using the app and accelerometer correctly?
- How should we check in with participants to see how they feel about the program?
- What are your ideas about keeping the program going after the grant ends?

**Table 1:** Focus group and key informant participants’ sociodemographic characteristics and relevant health behaviors (N=54).

Characteristics		Focus group (n=38)	Key informants (n=16)
Age (years), mean (range); SD (4.5)		49.6 (23-78)	44.1 (25-61)
**Sex, n (%)**			
	Male	12 (32)	7 (44)
	Female	26 (68)	9 (56)
**Marital status, n (%)**			
	Married	26 (68)	13 (81)
	Divorced or separated	5 (13)	—^a^
	Never married	4 (11)	3 (19)
	Widowed	2 (5)	—
	No response	1 (3)	—
**Education, n (%)**			
	Grade 12 or general educational development	4 (11)	1 (6)
	College 1-3 years	11 (29)	2 (13)
	College 4+ years	7 (18)	4 (25)
	Graduate school	16 (42)	9 (56)
**Work, n (%)**			
	Full time	22 (58)	13 (81)
	Part time	2 (5)	2 (13)
	Student or part time	2 (5)	1 (6)
	Homemaker	3 (8)	—
	Retired	7 (18)	—
	Unemployed or disability	1 (3)	—
	Student	1 (3)	—
**Financial status, n (%)**			
	Struggle to get by	6 (16)	1 (6)
	Struggle or about enough	1 (3)	—
	About enough	13 (34)	5 (31)
	More than enough	15 (40)	7 (44)
	No response	3 (8)	3 (19)
**Fruit and vegetable consumption, servings per day, n (%)**			
	1-2	17 (45)	5 (31)
	3-4	17 (45)	7 (44)
	5+	4 (11)	3 (19)
	No response	—	1 (6)
**Screen time, hours each day not including work or school, n (%)**			
	None	1 (3)	—
	1-2	16 (42)	8 (50)
	3-4	14 (37)	4 (25)
	5+	7 (18)	3 (19)
	No response	—	1 (6)
**Exercise, min per week of moderate to vigorous exercise,** **n (%)**			
	None	2 (5)	—
	1-60	1 (3)	—
	60-90	14 (37)	6 (38)
	91-119	7 (18)	—
	120-180	2 (5)	1 (6)
	181-240	4 (11)	5 (31)
	240+	8 (21)	3 (19)
	No response	—	1 (6)
**Do you use a smartphone, n (%)**			
	No	1 (3)	—
	Yes	35 (92)	15 (94)
	Sometimes	1 (3)	—
	No response	1 (3)	1 (6)

^a^Missing data, no response recorded.

### Data Analysis

The tape-recorded sessions were transcribed verbatim and immediately subjected to coding to determine the completeness and appropriateness of the questions and to ensure data saturation. Thematic analytic steps include close reading and rereading of transcripts, line-by-line coding, and codebook development [31]. In total, 2 members of the research team read all the transcripts to structure the codebook. During the second reading, 1 reader independently generated a list of codes that were crosschecked with each of the other analysts to produce coherent categories; the senior researcher approved the final codebook. We hand coded all transcripts, which, according to most qualitative standards, is considered more time consuming but just as appropriate as employing a software management system [32]. Moreover, hand coding is particularly well suited when template coding is used [33]. During the process of template coding, memos were developed to identify the relative frequency of the codes and the different contexts in which they emerged [34]. We assessed the relative frequency and variation in thematic presence among participants [35]. Themes that appeared across multiple participant transcripts are presented in the *Findings* subsection, with attention to commonalities across focus groups and key informant interview participants’ responses. We followed the steps for thematic analysis by Braun and Clarke [36], first familiarizing ourselves with the data; then embarking upon an iterative coding process for semantic content; and then searching for, reviewing, and naming themes. A second team member reviewed potential themes by checking for coherence with the selected extracts from the original transcripts. The final theme names and definitions were developed by 2 qualitatively trained researchers in collaboration with the first author as the writing process evolved. Research rigor was established via team analysis, prolonged engagement with the subject matter, and reflexivity.

### Rigor

We established research rigor through several approaches, including team analytic procedures, extensive reflexivity, peer debriefing, prolonged engagement in the community, and focal issues [36]. We used Lincoln and Guba’s conceptualization of qualitative standards of credibility (confidence in the *truth* of the findings, accomplished through prolonged engagement in the research environment and peer debriefing), transferability (whether findings apply to other contexts, established by memoing and case study development), dependability (demonstrating the capacity for the findings to be repeated and remain consistent, accomplished through engaging in inquiry audits), and confirmability (whether participants shape our findings rather than researcher bias or preconception, determined by maintaining an audit trail and engaging in reflexivity among the research team and participants) [37]. Peer debriefing included discussing core themes of the findings with community advisory board members to understand how and in what ways our findings resonated with their experience of community realities [3].

## Results

### Sample

#### Focus Groups

Table 1 includes the demographic information for the 38 focus group participants and 16 key informants. The average age (in years) of the focus group participants was 49.6 (range 23-78; SD 4.5). Most participants (26/38, 68%) were female, married (26/38, 68%), and nearly all owned a smartphone (35/38, 92%). Over half (22/38, 58%) of the participants worked full time, and nearly three-quarters (28/38, 74%) of the participants indicated that they have about or more than enough to make ends meet. Only under half (16/38, 42%) of the participants held a graduate degree. Most (22/38, 58%) of the participants reported consuming 1-4 servings of vegetables per day. One-fifth of the participants reported moderate or vigorous exercising 200 minutes per week or more, and over half (21/38, 55%) of the participants reported exercising between 60 and 120 minutes per week. Nearly 80% (30/38) of the participants reported having 1-4 hours of recreational (not related to work or school) screen time per day.

#### Key Informants

Participants included nearly equal number of males and females, most (13/16, 81%) of whom were married, had at least an associate degree (13/16, 81%), worked full time (13/16, 81%), *had about or more than enough to make ends meet* (12/16, 75%), and owned a smartphone (15/16, 94%). Most (12/16, 75%) of the key informants reported consuming 1-4 fruits and vegetables per day. Half of the participants (8/16, 50%) reported 1-2 hours of daily screen time not related to work or school, with most of the remaining participants reporting more screen time daily. One-third of the key informants (6/16, 38%) reported 60-90 minutes of moderate or vigorous exercise per week, whereas another third (5/16, 31%) reported 181-240 minutes of moderate or vigorous exercise per week.

### Findings

Focus Groups and Key Informant Interviews: As qualitative developmental research aims to obtain a holistic understanding of a phenomenon, findings from these 2 data sources are merged to present a cohesive response to the research question. From the focus group and key informant interview analysis, 3 major themes and subthemes emerged pertaining to perceived acceptability and feasibility of mHealth or health coaching interventions within rural communities. These themes were pervasive across focus groups and key informant interviews and included the following: (1) personal technology is feasible and desirable; (2) challenges persist in implementing mHealth lifestyle interventions in Appalachian communities; and (3) successful mHealth interventions should include personal connections, local coaches, and educational opportunities.

#### Personal Technology Is Feasible and Acceptable

The Appalachian residents in our study considered mHealth interventions feasible and, therefore, promising for 3 main reasons. First, the increased availability and use of smartphones, internet services, and other personal technology in rural areas support mHealth interventions. Participants indicated that most people in Appalachian communities use mobile technology. A fitness studio owner participating in a key informant interview commented on changing patterns of technology use he has noticed in his studio:

Fifteen years ago, the parents were watching what was going on the floor and now you can look back there and 80 percent of them are looking at their phones.

As the use of mobile technology has become more prevalent, awareness of the unique ways mobile technology can support fitness has also spread. For example, in rural areas, where lifestyle guidance can be difficult to find, mHealth interventions may provide personalized information for healthy living. Appalachian residents also recognized unique ways in which mHealth programs encourage personal accountability and awareness. An information technology expert suggested the following during a key informant interview:

I think it would be helpful for the app to use the data and encourage them. If the app can collect data, then you know you can set up different sections of communication through the app, so like a pop up in the app to say, “Hey you didn't reach your daily goal.”

Also commenting on how abundant exposure to mobile technology could enhance awareness and support healthy lifestyles, a key informant remarked:

This app, it’s a great thing because they’re always going to be staring (at it) especially if that app has notifications on it. It cues them to say, “Hey look at me for a minute.”

A focus group participant with a community organizing background noted:

The program has the potential to change lifestyles. It will make people more aware of screen time and being sedentary.

Another subtheme of feasibility and acceptability involves changing patterns of interaction that encourage the use of personal technology. Specifically, participants suggested that the app converges with an increasing preference for and ability to engage in programs on their own. A focus group participant explained:

Working individually has advantages because working in groups can be intimidating. People are more willing sometimes to do things individually rather than with a group.

She also noted the benefits apps have in facilitating communication:

Social media is a great way to communicate...People have shied away from phone calls.

A final subtheme of feasibility and acceptability involves enthusiasm for new approaches to lifestyle improvement, making mHealth interventions promising in rural Appalachian communities. Noting growing community awareness of chronic disease threats, participants indicated a greater openness to diverse, new opportunities for lifestyle improvement, including farmers’ markets, fitness centers, and personal technology. According to a focus group participant with an entrepreneurial and community organizing background, this openness lends itself to residents trying new approaches if they are locally based. Commenting on the feasibility of the mHealth or personalized health coaching intervention, the participant mentioned the following:

People would be very willing to do this. Most people in our area, there is a movement to eat better. The farmers’ markets are promoted and there’s interest there. People are becoming a lot more conscious of their health. There’s been enough national media talking about increases in cancer; people are concerned and would be willing.

A key informant who has struggled with fitness and diet her entire adult life noted similar perspectives among fellow congregants in her large rural church:

People are getting more interested in physical activity and want to do better. Being African American, I know I have many friends who are concerned with blood pressure and diabetes.

A physical therapist key informant who helps people become more physically active agreed:

I think people will be willing (to participate). Many individuals want to get in shape and be healthier, but they don’t know where to start.

#### Challenges to mHealth Lifestyle Interventions in Appalachia

Despite the potential utility of mHealth lifestyle interventions, participants described numerous challenges that must be overcome to ensure a successful mHealth or personalized health coaching intervention. Participants expressed guarded enthusiasm for such interventions, explaining that challenges exist in the feasibility and acceptability of such innovation at all levels. Consistent with the socioecological framework [38], these challenges include individual-, intrapersonal-, community-, and system-level factors. At an individual level, limited familiarity with healthy lifestyle practices poses a barrier to successful mHealth interventions. Reflecting on her years of teaching youth about healthy eating as a food and consumer science educator, a key informant explained:

It is challenging to access a wide variety (of fresh produce), but I find that most people don’t even eat the basics. Apples, oranges, bananas...People just don’t eat fruit. When I was teaching culinary at the high school, I would do taste tests on fruit. I might have a star fruit and a pomegranate, and I would let kids taste different things and they never knew they liked fresh pineapple. They never knew they liked cantaloupe because they had never tasted or never had it before. So, a lot of times I think it is just the lack of knowledge. And they have never been introduced to that food.

A focus group member who works in the fitness field voiced a similar concern about unfamiliar food items:

People just don’t know what to get or how to fix it. I think you have to watch with people in this area about how you present things as well. They are easily made to feel like they didn’t know that so they must be dumb.

In the realm of technology, another focus group participant explained that limited familiarity with technology might pose barriers to successful mHealth programming:

Smartphone access is a barrier, especially with elderly participants. Elderly participants will also need extra training on the app.

Compounding the effects of individual barriers, intrapersonal barriers, including a legacy of unhealthy habits reinforced over generations, may limit the success of mHealth programming among rural residents. A key informant explained that many people:

were raised that way just like their parents were raised that way. You know my grandmother...there was more grease in the food than there was food.

A key informant with a health promotion background emphasized the power of tradition to shape peoples’ lifestyle:

But if it’s been a generational thing. If your parents didn’t exercise you don’t exercise, you’re probably not going to exercise.

She explained how cultural patterns, shaped in part by the geographical landscape, also limit community members’ activities:

I think the activity level, especially here in the mountains, people seem to be pretty dormant in winter months is kind of like hibernation and like the bears that hibernate. And as soon as the temperatures start increasing you see more people out like in parks at basketball court, you have little league, basketball and soccer. You just see people out more active, but in the winter months I think that would be a really hard time to be successful with the project.

In many rural areas, community-level barriers, including cultural traditions of unhealthy choices, intersect with significant and persistent resource scarcity, potentially limiting the success of mHealth or personalized health coaching interventions. A key informant explained:

Things like economics, the health issues that we have here in eastern Kentucky are a byproduct of financial burdens. The old joke is nutrition is more expensive than drugs.

A young father participating in a focus group noted:

the closer you get to the poverty line, the less people tend to be worried about their nutrition and health.

Speaking specifically about the impact of the financial burden on access to technology, the information technology key informant explained:

The only concern I would have (regarding mHealth interventions) is that the iPhone is gonna be more expensive for the most part. If you’re looking at a Samsung Galaxy S 10 and iPhones they’re clearly very compatible. But if you can look at some pretty low-end android phones that are pretty cheap versus you know a brand-new iPhone. So, I don’t know if the app is developed for both. I think it would be very helpful if it was.

Although improving throughout the region, internet access remains another barrier to mHealth intervention success. As the fitness studio owner, a key informant explained:

There are some places that have quote unquote “bad spots” in the area. So, yes that could definitely be a hurdle. It seems to be improving but, it’s an on-going joke: having a cell phone in Kentucky is an oxymoron because it’s useless. It used to be useless about half the time and 80 percent of the time but now it’s about 20 percent of the time, so it’s getting better.

An older focus group participant who works with community food access agreed:

Access to a smartphone that can handle the app; availability of access to Wi-Fi connection I think that’s one of our biggest problems around here is the Wi-Fi connection. There are parts of this county where you cannot get a signal. Therefore, some of the data may not download. You’d have to go somewhere where it does.

A key informant added his concerns regarding people’s access to cellular phone services:

I am not sure if everyone has the cellphone coverage needed to be able to use the app accurately...Cellular reception is terrible here. According to the coverage map we should have coverage, but we don’t at our house.

#### mHealth Implementation Requires Personal Connections, Local Coaches, and Educational Opportunities

Participants indicated that the implementation of mHealth programming, though challenging, could be made feasible and acceptable by ensuring extensive local engagement and staffing. First, the participants emphasized personal connections to help support each other. Reflecting on her experience coordinating programs to help people live healthier, a focus group participant observed:

People do better walking together with someone rather than by themselves. If they have someone to share with, they may help each other along the way.

Another key informant reflected:

When you do it by yourself, most people will fail. But if they have a coach, if they have somebody else to work with and talk to and things like that and that accountability is there where you are not just letting yourself down and you don’t let your friend down.

With novel communication apps continually available, allowing friends to communicate constantly using video, audio, and text, mHealth interventions may increase motivation by “connecting (participants to) others using social media or a member chat within the app. They can give each other feedback and support. If they have a partner, things are much easier and they are given that accountability.” This connectivity may be especially important for older adults who are less familiar with technology; however, numerous participants advocated revising the upper age limit (65 years) as an increasing number of healthy older adults embrace this technology.

Participants also viewed local coaches to enhance relational support. A focus group participant explained that program facilitators must “be knowledgeable about the area. Local people will be more effective.”

A key informant elaborated:

Many people are leery of outside groups coming in and telling them what they need to do or how they need to do it.

A focus group participant concurred:

Nonlocals could be seen as condescending.

Though she also noted:

Locals could be a barrier just by knowing or hearing about them. Locals sometimes have “baggage”...The focus should be on finding the person who can offer the best services.

According to a key informant:

Using local people will get rid of that “outsider” feel.

Reflecting on his years coaching children and adults, the key informant fitness studio owner shared:

I think that there would be a lot of people locally that could be qualified to do so (coach within mHealth programming) with the right training and through understanding how to coach people...If you can get a coach that can look at someone, make eye contact, and see that inner stride inside of them and figure out what it takes to pull that out of them instead of just being a motivational speaker. Yeah, I mean you can do that here as well as you can do that anywhere.

Finally, reflecting on suboptimal health traditions common in their community, participants considered mHealth interventions that increased participants’ knowledge about healthy living practices as the most promising. A key informant explained the importance of providing education with accountability:

If you had something to guide them along the way with nutritional facts and just basic physical activities it would be very beneficial. I think people will be more prone to go through with it. A lot of people are too scared to start. By getting the one-on-one alone time it will help people to not give up and they will be more likely to follow through. Calorie intake and information that can be given through the app will be helpful as well.

A focus group participant also shared how learning about positive results would motivate him to maintain a healthy lifestyle:

Understanding that OK well if I feed my body properly and I get some calories burned in the right way, I may see some improvements in the way I look physically, or I may be influenced in the way I feel physically. Just knowing that there are benefits to it and understanding those benefits to it.

## Discussion

### Principal Findings

We examined Appalachian residents’ perspectives on the feasibility and acceptability of a mHealth intervention that promotes healthy diet and active lifestyle. We specifically focused on the mHealth component of the intervention because other components (personalized health coaching, accelerometer use, and economic incentives) have been extensively described and characterized in this population. Focus groups and key informant interviews revealed that rural Appalachian residents consider such interventions promising, although persistent resource scarcity raised some concern about feasibility. Below, we discuss 3 insights emerging from this work and their implications for future research.

First, participants described the untapped but vast potential of mHealth programming in rural contexts, noting that most of their neighbors have sufficient access to and experience with using app-based programming to support the implementation of mHealth lifestyle interventions. It is important to emphasize that MBC2 consists of elements other than simply the technology; for example, participants expressed strong support for personal health coaching, which is likely the most critical part of the program. Our results corroborate previous studies in Appalachia demonstrating widespread internet access [16] and the feasibility and acceptability of mHealth [39].

Second, despite the study participants’ enthusiasm for and comfort with using app-based programming, sparse resources remain a barrier to all lifestyle intervention success. Specifically, study participants described how limited access to material goods (eg, fruits and vegetables and physical fitness venues) pose barriers to the potential success of lifestyle interventions in rural communities. Although the use of the internet is common among most rural adults (78%), and 66% of rural adults have a smartphone [16], access to smartphones varies greatly by age, socioeconomic status, and geographic region [15]. It is possible that scale-up of the MBC2 or other mHealth or personalized health coaching interventions may provide less benefit to the most vulnerable populations in the region. However, given the widespread and pernicious health inequities, improvement across all population segments, even the relatively well resourced, is warranted. Moreover, given that over half the population currently uses personal technology and rates of use are increasing, it seems provident to begin testing and improving this intervention in preparation for greater technology saturation.

Third, although mHealth interventions may provide rural Appalachia residents access to advice and guidance for living a healthy lifestyle, limited access to high quality, affordable foods, and physical activity resources impede rural residents’ ability to act on this guidance and support [6]. As participants acknowledged these barriers, they also provided nuanced guidance for tailoring mHealth interventions for success in vulnerable rural communities. Participants desired mHealth or personalized health coaching interventions that emphasize personal connections with friends and locally appropriate personnel, organizations, and lifestyle guidance. Consistent with other multiple health behavior interventions, most of our participants preferred lifestyle interventions that combined in-person and technology-based methods [14]. For example, many participants advocated for face-to-face group-based activities, a departure from MBC2. Additional recommended adaptations included local health coaches and integrating local organizations and resources into this mHealth or personalized health coaching intervention. These recommendations do not require dramatic modification and converge with Appalachian traditions of self-reliance and mutual aid and support [5,8].

### Limitations

Although this study identified important findings regarding the acceptability and feasibility of a mHealth or personalized health coaching intervention in rural communities, we acknowledge several limitations. First, this study was conducted solely in Appalachia; thus, the findings of this project may not be applicable to other contexts. Second, with the rapid advancement of technology, these findings will require frequent updates. We suspect that our findings will be increasingly relevant as more Appalachians use personal technology. Third, our participants had higher incomes, more education, and tended to engage in physical activity more than others in the region. Although our participants’ socioeconomic status is likely to be similar to that of future intervention participants, such differences may raise questions about accurately characterizing the entirety of the local community. We would counter that given the close integration and connection among community members, even participants with slightly higher socioeconomic status and more optimal health behaviors have health profiles and behaviors that would be improved by this intervention and thus have relevant perspectives. In addition, purposive sampling of key informants necessarily involves more professionals, who may slightly differ in their backgrounds from the general population. Finally, the modest sample size precludes generalizability and diversity.

Despite these limitations, this study provides insights into the promising potential of mHealth or personalized health coaching interventions to support healthy lifestyle among residents of underserved rural communities. Future studies are needed to determine ideal strategies for enhancing lifestyle programming through connections to community-based resources.

## Abbreviations

CFIR: Consolidated Framework for Implementation Research
MBC2: Make Better Choices 2
mHealth: mobile health
RUCA: rural-urban commuting area
